# Guardian of the Genome: An Alternative RAG/Transib Co-Evolution Hypothesis for the Origin of V(D)J Recombination

**DOI:** 10.3389/fimmu.2021.709165

**Published:** 2021-07-28

**Authors:** Iryna Yakovenko, Jacob Agronin, L. Courtney Smith, Matan Oren

**Affiliations:** ^1^Department of Molecular Biology, Ariel University, Ariel, Israel; ^2^Department of Biological Sciences, George Washington University, Washington, DC, United States

**Keywords:** RAG1, RAG2, RSS, transposons, immunological big bang, terminal inverted repeats, guns for hire, adaptive immune system evolution

## Abstract

The appearance of adaptive immunity in jawed vertebrates is termed the immunological ‘Big Bang’ because of the short evolutionary time over which it developed. Underlying it is the recombination activating gene (RAG)*-*based V(D)J recombination system, which initiates the sequence diversification of the immunoglobulins and lymphocyte antigen receptors. It was convincingly argued that the *RAG1* and *RAG2* genes originated from a single transposon. The current dogma postulates that the V(D)J recombination system was established by the split of a primordial vertebrate immune receptor gene into V and J segments by a *RAG1/2* transposon, in parallel with the domestication of the same transposable element in a separate genomic locus as the RAG recombinase. Here, based on a new interpretation of previously published data, we propose an alternative evolutionary hypothesis suggesting that two different elements, a RAG1/2 transposase and a *Transib* transposon invader with RSS-like terminal inverted repeats, co-evolved to work together, resulting in a functional recombination process. This hypothesis offers an alternative understanding of the acquisition of recombinase function by RAGs and the origin of the V(D)J system.

## Introduction: V(D)J Recombination and RAGs

An outstanding feature of the jawed vertebrates is their adaptive immune system that is capable of recombining gene segments to create a diverse repertoire of immunoglobulins (Igs) (1, 2) and T cell receptors (TCRs) (3). After the primary response to a specific pathogen, the adaptive immune system mounts an enhanced secondary response to subsequent encounters with that pathogen, which is the basis for immunological memory. Sequence variation of the antigen-binding sites in the Igs and TCRs is essential for the host immune system to recognize and destroy the extensive array of pathogens before any irreversible damage takes place. Hence, the mechanisms to diversify the Ig family repertoire are of significant advantage for the fitness of higher vertebrates.

**Figure 1:**
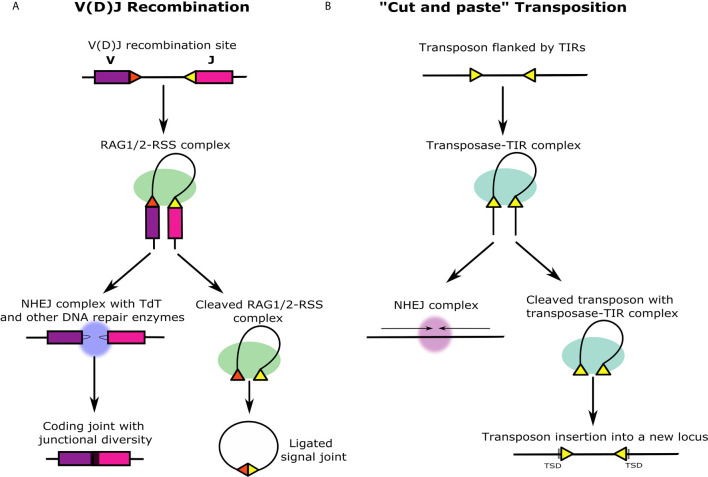
Similarities and differences between V(D)J recombination and ‘cut and paste’ transposition. **(A)** V(D)J recombination occurs at the immune gene loci in differentiating lymphocytes during early T and B cell maturation stages. The RAG1/2 protein complex (green) binds to two asymmetric RSSs (yellow and red triangles) flanking *V*, *D*, and *J* gene segments (in this illustration, the *D* segment is not shown). The DNA double helix bends and folds into the recombination synaptic complex based on the selected RSS pair. Next, RAG1/2 introduces a nick at the intersection between each RSS and the coding gene segment that leads to the formation of closed DNA hairpins on the coding segments, and blunt, 5′ phosphorylated RSS ends at the signal ends that remain associated with the RAG1/2 complex and are ligated together forming a signal joint. The signal joint circle is deleted from the genome. Before ligation, the coding ends are subjected to further diversification by DNA repair enzymes together with TdT (blue) that generate junctional sequence diversity (black region between purple and pink gene segments). **(B)** ‘Cut and paste’ transposition starts similarly to V(D)J recombination with the transposase enzymes binding to the TIRs flanking the ends of the transposon (yellow triangles). Analogous to the beginning of the V(D)J recombination, the DNA double helix bends and folds into a transposition synaptic complex. The transposase makes double-stranded breaks in the DNA, and the transposon is entirely excised including the TIRs. The genomic location from which the transposon is excised is immediately ligated by NHEJ mechanism. Unlike the excised V(D)J signal joint circle that is lost from the genome, the excised transposon with the transposase-TIR complex creates a double-stranded break in a different region in the genome and integrates into the target site. This activity generates target site duplications (TSDs) on both sides of the integrated transposon that are formed similarly in RAG transposition events.

**Figure 2:**
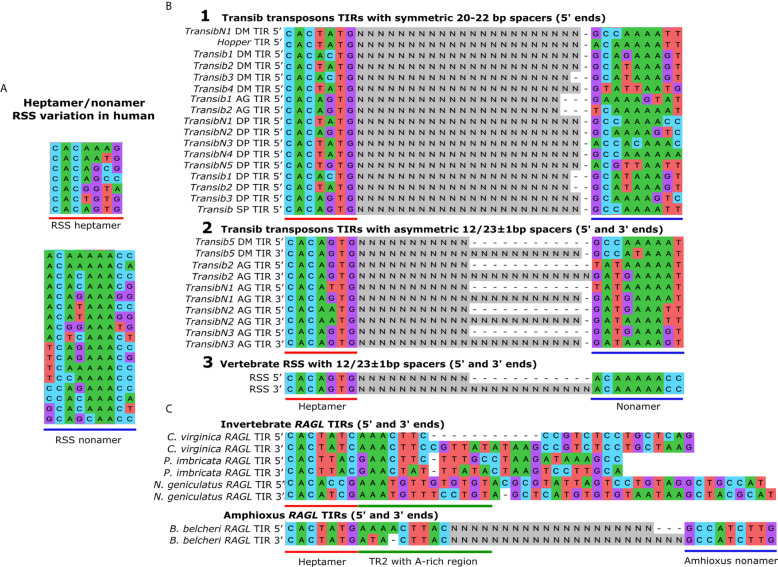
The TIRs in the *Transib* transposon family are structurally similar to the RSSs in the *Ig* family. **(A)** RSS variation in human *Ig* loci. The conserved nucleotides in the RSSs in the heptamer (underlined in red) and the nonamer sequences (underlined in blue) are necessary for efficient and precise V(D)J recombination. The key feature of the heptamer is the conserved CAC consensus sequence, and the most common heptamer sequence in *Ig* loci is CACAAAG. The key feature of the nonamer is the core A-rich region, and the most common sequence is ACAAAAAG. Sequences were obtained from the IMGT database (http://www.imgt.org). **(B)** *Transib* TIRs compared to RSSs. Alignment 1. The heptamer and nonamer-like sequences are shown for the *Transib* transposon TIRs with symmetric 20-22 bp spacers (indicated as Ns). Alignment 2. The 5ʹ and 3ʹ TIR sequences of *Transib* family members with 12/23 asymmetries include *Transib* 5 from *Drosophila melanogaster* and *Transibs* 2, *N1*, *N2* and *N3* from the malaria mosquito, *Anopheles gambiae*. Alignment 3. The most common human RSS sequences are shown that surround *Ig* family gene segments. TIR sequences in **(B)** were obtained from (8). **(C)** *RAGL* TIRs in invertebrates. Asymmetric TIRs are associated with the *RAGL* sequences from the eastern oyster, *Crassostrea virginica*. Symmetric TIRs surround *RAGL* sequences from the pearl oyster, *Pinctada imbricata*, and the robber fly, *Neomochtherus geniculatus*. Asymmetric 27/31 TIRs are present in the *protoRAGL* from amphioxus, *Branchiostoma belcheri*. All sequences include a 5ʹ *RSS*-*L* heptamer sequence and a partially conserved A-rich transposon region 2 (TR2) located towards the 3ʹ half of the heptamer. No *RSS*-*L* distinctive nonamers have been identified in *B belcheri*. TIR sequences in **(C)** were obtained from (9).

Igs are composed of heavy and light chain subunits, and their unique antigen-binding structures are accordingly determined by the recombination of either two or three types of gene segments. The heavy chain variable domain is encoded by variable (V), diversity (D), and joining (J) segments, while the light chain variable domain lacks the D segment (4, 5). TCRs have an analogous heterodimer structure with protein chains containing variable domains that are similarly encoded by different combinations of either *VDJ* or *VJ* segments (6). The recombination of *VDJ* or *VJ* gene segments, commonly defined as V(D)J recombination, is facilitated by a complex of two enzymes that are encoded by the recombination activating genes (*RAG1* and *RAG2*) (7). The RAG1/2 complex recognizes and binds to recombination signal sequences (RSSs) that flank the *V(D)J* gene segments (**Figure 1A**). RSSs function as terminal inverted repeats (TIRs) and are composed of semi-conserved heptamer and nonamer sequences separated by a spacer region of either 12 or 23 base pairs (bp) (**Figure 2A**). According to the 12/23 rule, V(D)J recombination can only occur asymmetrically, based on the pairing of RSSs with 12 and 23 bp spacers (10). This system maintains the specificity of the recombination process and prevents the gene segments from recombining incorrectly and the *Ig* loci from recombining with other *Ig* or *TCR* loci. Recombination between gene segments is initiated early in the development of lymphocytes by the RAG1/2 complex bound to the RSSs. The complex nicks DNA at the 5′ end of the RSS heptamer at the junction with the coding gene segment. This allows the free 3′ OH group to attack the phosphodiester bond on the opposite strand in a transesterification reaction that forms covalently closed hairpins at the ends of the coding segments and blunt-end double-strand breaks at the ends of the RSS heptamers (11). The RSS ends are ligated head-to-head to form the signal joint in the excision circles. On the other hand, the coding ends are subjected to a modified non-homologous end joining (NHEJ) process that creates additional diversity within the V(D)J coding junction. Orchestrated by DNA repair enzymes, the process is augmented by terminal deoxynucleotidyl transferase (TdT) that adds random nucleotides to the junction before nucleotide annealing and DNA backbone ligation of the two coding ends in a head-to-tail fashion (**Figure 1A**) (12). These diversification processes give rise to an impressive repertoire of Igs and TCRs. It was estimated theoretically that the diversity of the human Ig and TCR proteins might reach between 10^11^ to over 10^18^ variants. Accelerated single point mutations of the *Ig* variable exons during B cell affinity maturation may further increase the *Ig* diversity to an incomparable estimate of 10^52^ possible variants (13).

## The Currently Accepted Transposon/Split Receptor Gene Hypothesis for the Origin of theV(D)J System

The RAG-based adaptive immune system (AIS) has been estimated to have emerged in jawed vertebrates 450-500 million years ago. Its appearance has been referred to as the immunological ‘Big Bang’ because of the relatively short evolutionary time over which it evolved (14). It was speculated that two primary events were necessary for the emergence of an AIS - the appearance of *RAG* genes and two rounds of whole-genome duplications, that provided multiple copies of genes required for accelerated AIS evolution, including the *Ig* gene family. For example, four paralogous gene regions that encode the major histocompatibility complex (MHC) proteins and complement proteins, which are critical elements of the vertebrate AIS, are likely the result of such duplications [reviewed in (15)]. Because RAG-mediated V(D)J recombination is the fundamental process for jawed vertebrate combinatorial immunity, it became the focus of research on the evolution of the vertebrate AIS.

Almost since the discovery of the V(D)J recombination system, it was proposed that the structure of the loci encoding the antigen receptors might have been attained by the insertion of a particular ‘alien seed’ into a primordial vertebrate antigen receptor that split it into *V* and *J* segments (4). The term alien seed has been used to describe a possible ancient transposon that was inserted, of which only its TIR sequences (*i.e*., the RSSs) have remained throughout evolution. This hypothesis was later extended to include the formation of a *D* segment as a result of the duplication events that led to formation of the VDJ segments clusters (16). It was also noted that the V(D)J recombination process bears fundamental features of DNA cut-and-paste transposition [*e.g* (17)] (**Figure 1**). The RAG protein structure contains a DDE catalytic motif, critical for RAG1 function (18), which is also a key motif in certain integrases and transposases (18, 19). Moreover, RAG1 and RAG2 work together as a transposase *in vitro*, that is capable of excising a DNA segment bounded by RSSs and, in few cases, inserting it into a different location in the DNA (20, 21). Short DNA duplications that accompany these *in vitro* transpositions on both sides of the insertion are highly reminiscent of the ~ 5 bp target site duplications (TDS) that is a hallmark of DNA transposons (22). The function of the RAG recombinases, together with the tight physical linkage between vertebrate *RAG1* and *RAG2* genes, implied that the two genes might have acted once as a single transposon (23, 24) (*RAG1/2* transposon).

To explain the evolutionary appearance of the vertebrate RAG recombinase simultaneously with its *V(D)J* targets, it was suggested that the *RAGs* and the split antigen receptor event were derived from at least two insertions of the same *RAG1/2* transposon into the germline early in the evolution of the vertebrate lineage (16, 25–27). Another version of the transposon/split receptor gene hypothesis suggest that the original RAG1/2 recombinase and the split of the immune receptor gene derived from different but similar mobile genetic elements that were originated from a common transposon ancestor [*e.g* (28, 29)]. To gain credence for the theory, researchers searched invertebrate genomes for such a transposon with core regions encoding both RAG1 and RAG2 proteins. Indeed, the supposed missing link or an active ‘living transposon fossil’ was identified in the lancelet, *Branchiostoma belcheri*, and was termed ‘*protoRAG’* (30). This finding seemingly enforced the existing transposon/split receptor gene hypothesis (8), which has become the sole interpretation for the ample findings that have accumulated in this field. However, a few major questions regarding the process remained unanswered by the transposon/split receptor gene hypothesis. Perhaps the most important question is what were the evolutionary selection forces that drove the formation of this rather complicated mechanism? Despite this gap in understanding, no alternative hypothesis has been proposed.

## RAG Origin and Evolution

**Figure 3:**
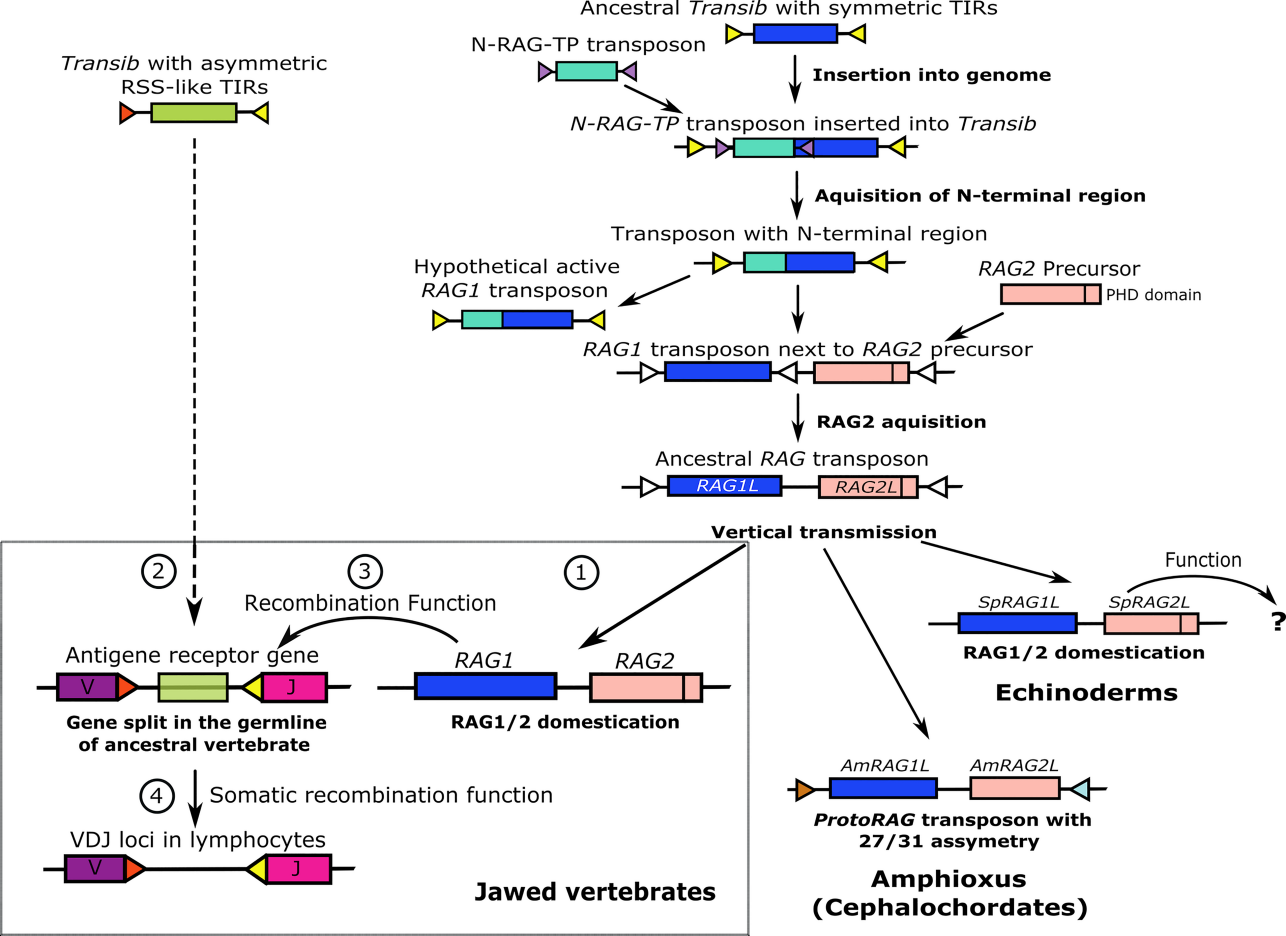
A hypothesis for *RAG* evolution; an alternative to the transposon/split receptor gene model. The evolution of *RAG* genes begins as an ancestral *Transib* transposon with symmetric TIRs. Insertion of an *N-RAG-TP* transposon and recombination with the ancestral *Transib* gave rise to a new transposon with all the core features of an extant *RAG1* gene, including identical TIR sequences and the N terminal region. An active *RAG2* precursor GE was inserted close to or inside the *RAG1L* gene that became an ancestral hypothetical *RAG1/2* transposon that was mainly further transmitted vertically. In amphioxus, the *protoRAG* transposon lost its PHD domain but maintained its transposition activity based on 27/31 asymmetric TIR spacers. In sea urchins, the *RAG1/2L* genes were domesticated and may have gained function, which is currently unknown. According to our guardian of the genome hypothesis (lower left box), *RAG1/2* was also domesticated early in the jawed vertebrate lineage to protect vertebrate genomes from insertion and excision of harmful TEs (labeled as 1 in the box). Later, a *Transib* transposon with 12/23 asymmetric TIRs entered and survived inside a jawed vertebrate antigen receptor exon, splitting it into *V* and *J* segments (2 in the box). As an ex-transposon endonuclease, the RAG1/2 machinery was co-opted and trained to excise the asymmetric TIR-flanked *Transib* transposon threat in the *V*(*D*)*J* receptor loci. Fortuitously, the excision process in the *V*(*D*)*J* loci provided an immunological advantage and therefore the RAG1/2 complex gained its V(D)J recombinase function in the germline (3 in the box). Eventually, under selective pressure, the V(D)J machinery was selected evolutionarily to work primarily in the lymphoid cell lineages (4 in the box).

In parallel with investigations to define the functions encoded by the vertebrate *RAG* genes, efforts have been made to trace possible transposon relatives of the *RAG* genes that would shed light on their evolutionary origin. Kapitonov and Jurka (8) identified several DNA transposons of the Transib superfamily in invertebrates with predicted transposase cut-and-paste function. The transposase contains a functional core region of about 600 amino acids that is highly similar to the core region of vertebrate RAG1 and includes the DDE motif essential for the RAG recombinase catalytic activity (8). Among all of the DDE transposases investigated, the *Transib* family shows the highest sequence similarity to *RAG1*. Furthermore, these transposons exhibit 5 bp TSDs (8) that are also observed upon experimental transpositions with RAG1/2 (20). This led to the conclusion that the ancestry of the *RAG1* gene lies within the *Transib* transposon family (8). Although some *Transib* family members are similar to *RAG1*, none include an N terminal region that is present in *RAG1*. The clue for the origins of the N terminal region in *RAG1* came with the discovery of a transposable element (*N-RAG-TP*) in the sea slug, *Aplysia californica*, that is composed entirely of the RAG1 N-terminal-like sequence (31). This finding led to the assumption that the complete *RAG1* structure was likely derived from the recombination between a *Transib* and the *N-RAG-TP* transposon. We suggest that the *N-RAG-TP* transposon was inserted into the 5′ end of the *Transib* sequence and not vice versa, keeping the original *Transib* TIRs on both sides of the recombined sequence without interrupting the transposase function (**Figure 3**).

Solitary *RAG1* orthologues (*RAG1L* genes) that encode the N terminal domain are present in invertebrates, and vertebrate RAG1 may function independently of RAG2 (23, 32). Therefore, it was assumed that the *RAG1* and *RAG2* genes were joined together in the *RAG1/2* locus after the primordial *RAG1* gained the region encoding the N terminal domain (27) (**Figure 3**). While it is not essential for transposition, the RAG2 protein has an important function in the precision of RAG1/2 recombinase activity. The RAG2 core consists of a six-bladed beta-propeller (WD40 repeat) that interacts with both RAG1 and the DNA of the coding segment next to the heptamer (33). The non-core region of RAG2 consists of a specialized zinc finger or plant homeodomain (PHD), which binds to the N terminal tail of methylated histone 3 (H3K4me3) and is required for correct interaction with the open chromatin during recombination (34). A functional PHD domain is also encoded by *RAG2* from the sea urchin, *Strongylocentrotus purpuratus* (*SpRAG2*) (35), but is absent from the invertebrate amphioxus *protoRAG* transposon that does not have recombinase activity (30) (**Figure 3**).

The *RAGL* genes are found in multiple invertebrate deuterostomes, including echinoderms (36), cephalochordates (30), and hemichordates (acorn worms) (29) and were recently detected in protostomes, including oysters, mussels, ribbon worms, and even in the non-bilaterian cnidarians (9). These *RAGL* genes come in all flavors: *RAG1/2* pairs with intact or partial TIRs or without TIRs, unlinked *RAG1L* genes and *RAG2L* genes, as well as *RAG* pseudogenes. However, despite considerable efforts, *RAGL* genes have not been identified in jawless vertebrates or urochordates (tunicates) (9). This evolutionary gap may be the outcome of either horizontal gene transmission or loss in certain phylogenetic groups based on conventional vertical transmission (36). The horizontal transfer hypothesis, to its extreme, suggests that an ancient *RAG* transposon ‘jumped’ several times from some invertebrates into common ancestors of cephalochordates or jawed vertebrates and only later was domesticated in jawed vertebrates (37). However, while the amphioxus *protoRAG* transposon is likely to transpose *in vivo* (30), there is no evidence for inter-species transposition of the *RAGL* sequences. To the contrary, a recent analysis shows that the *RAG* sequence phylogeny is gradual and directional rather than patchy (present in some clades and absent in others), supporting a more conventional evolutionary path that relies on vertical, not horizontal *RAG* gene transmission (9). This hypothesis suggests that the *RAG1/2L* pair was possibly present in its current form in most metazoan lineages and may have been lost in the lineages of tunicates and jawless vertebrates. In any case there is no evidence that the V(D)J recombination system arose at any time earlier than the vertebrate lineage. Nevertheless, the necessity of generating sequence diversity among immune genes in tunicates and jawless vertebrates is not doubted, and therefore, the absence of V(D)J recombination may have driven the evolution of the other diversification mechanisms in these phylogenetic groups. An example for such mechanism is the copy choice mechanism of the variable lymphocyte receptor (*VLR*) gene family in jawless vertebrates that generates significant sequence diversity in the encoded antigen receptors and relies on the function of activation-induced deaminases (AIDs) rather than RAGs (38–41). We would like to note that the answer to the *RAG* transmission question still remains, and it is possible that both transmission types took place.

## The 12/23 Recombination Asymmetry and the ‘Transib Seed’

One of the critical elements in the V(D)J recombination system is the unique 12/23 bp asymmetric RSSs, that are equivalent to transposon TIRs. The 12/23 asymmetry rule appears to be more essential for the accurate execution of V(D)J recombination than the spacer sequences themselves that are highly variable (42). The 12/23 RSS asymmetry is only recognized when the RAG1 recombinase is complexed with RAG2 in jawed vertebrates. Although mouse RAG1 alone is capable of mediating V(D)J recombination in the absence of RAG2, its recombination efficiency is reduced significantly. Moreover, RAG1 alone does not show a preference for asymmetric 12/23 RSSs compared to symmetric 12/12 RSSs (23). Consequently, the possibility should be considered that the ancestral RAG1 gene might have originated from an ancestral Transib transposon with symmetric TIRs (**Figure 3**), similar to some existing *Transib* transposons (**Figure 2B**, alignment 1). Furthermore, recombinant sea urchin SpRAG1 (rSpRAG1) and rHzTransib, a functional *Transib* transposons from the corn earworm moth, *Helicoverpa zea* (43), both mediate V(D)J recombination through mouse 12/23 RSS sequences when combined with mouse RAG2. However, when rSpRAG1 is co-expressed with sea urchin recombinant rSpRAG2, it does not show recombination activity (23).

The above observations may suggest that V(D)J recombination, which relies on asymmetric 12/23 TIRs, dependent on the more recent evolution of RAG2 in the vertebrate lineage (23). Additionally, many of the *RAGL* transposons in early bilaterians, if they have transposase activity, probably rely on the ~15-17 bp TIRs that include a heptamer with an A-rich region (termed transposon region 2; TR2) and show no preference for spacer asymmetry (**Figure 2C**) (9). The only asymmetric invertebrate TIRs found are those that flank the *protoRAG* in amphioxus with 27/31 bp spacer asymmetry, which is very different from that of the *V*(*D*)*J* RSSs (44). This gap challenges the senario in which the antigen receptor gene was split into *V* and *J* segments by an ancestral hypothetical *RAG* transposon as proposed by the current hypothesis.

Remarkably, the only known transposition that incorporates 12/23 asymmetry other than RAG-based V(D)J recombination is found in some *Transib* transposon family members (**Figure 2B**, alignment 2) (8). This type of TIR asymmetry surrounds few *Transib* transposons and have almost perfect 12 or 23 ± 1 bp spacers with heptamer and nonamer sequences of which some are identical to those of the canonical RSSs in higher vertebrates (**Figure 2B** alignment 3). The probability that such sequence match between the RSS and *Transib* TIR termini occurred by chance is less than 10^-3^ (8). When including the match in the asymmetric spacer sizes of the 5ʹ and 3ʹ TIRs, the resemblance seems too great to be a coincidence. We propose that this unique asymetrical TIR structure has a functional role not only for the RSS mediated recombination, but also in *Transib* transposition as is known for other TIR transposons (44, 45). In light of the above, we suggest that the alien seed, which entered the primordial antigen receptor gene in the jawed vertebrate lineage, was not an ancestral *RAG* transposon as the current hypothesis suggests but may have been a *Transib* transposon with asymmetric 12/23 RSS-like TIRs similar to currently existing *Transib* transposons. This transposon insertion scenario is supported by the argument that the *Transib* transposons are smaller than the *RAG* genes and are more likely to have horizontal transposition capabilities based on their phylogenetic distribution (22). It is noteworthy that the *Transib* transposons are absent from the genomes of all vertebrates (8). This fact seems to challenge our suggested hypothesis. However, as discussed below, evolutionary forces involved in *RAG* domestication may explain, at least in part, the absence of *Transib* transposons from vertebrate genomes.

## Transposon Recruitment for Host Defense

Selfish mobile genetic elements (MGEs), including viruses and transposons or TEs, frequently invade genomes of organisms from all kingdoms (46). Most TEs are site-specific nucleases that function by incorporating their DNA into the genome (47, 48). Therefore, TEs may impact genomic integrity and cellular function, and thus may be harmful or lethal to the host [reviewed in (49)]. To resist an assault by TEs, successful life forms have developed complex defense mechanisms such as DNA methylation of the transposon-containing regions and sequence specific RNA degradation of TE transcripts (50, 51). Some organisms have recruited TEs to combat other TEs. In these cases, TEs have been incorporated into host genomes and domesticated or co-opted to function in host defense. The ‘guns for hire’ theory (52) addresses the question of how TEs could become the guardians against invaders through gradual mutations, immobilization, and domestication. Although domesticated TEs are widespread across many taxa, it remains unclear how TE domestication starts and proceeds (45, 53, 54). The CRISPR-Cas adaptive immune system of archaea and some bacteria is an example of co-option of TEs in prokaryotes for defense against bacteriophage infection (55). The adaptation module plus both varieties of effector modules in CRISPR-Cas are considered to have evolved from either transposons or transposon-like modules (56). Other examples for domesticated TEs for protection and/or development include the following: a) Recruitment of the *PiggyBac* transposon in ciliates, which removes TE-associated non-coding internal eliminated sequences (IES) (57, 58). b) Bacterial XerC/XerD and archaeal XerA recombinases that were potentially recruited from a range of protective MGEs and function in site specific resolution of circular chromosome dimers (59, 60). c) Piwi-interacting RNAs (piRNAs) that are encoded by small TEs and are present of many eukaryotes including mammals. The piRNAs associate with a PIWI nuclease mostly in germline cells that functions in cleavage and silencing of complementary TE transcripts (61). d) Finally, *gag, pol* and *env* genes in humans originated from endogenous retroviruses and have been co-opted to combat other retroviruses [reviewed in (62)]. Hence, the proteins encoded by TEs should be viewed as potential offensive weapons of the host in the evolutionary arms race between hosts and their DNA parasites.

## RAG Domestication and the Alternative RAG/Transib Co-Evolution Hypothesis

How the *RAG* transposon was domesticated to function as a recombinase has remained a major question for understanding the origin of V(D)J recombination (16). The current transposon/split receptor gene hypothesis does not provide an explanation for the domestication of the *RAG1/2* transposon prior to the formation of the whole V(D)J system. It assumes that both processes occurred simultaneously. This concept can be challenging because of the separate locations of the *RAG1/2* locus itself (chromosome 11 in humans), versus the RAG recombinase targets - the *VJ* loci of the kappa and lambda light chain genes (chromosomes 2 and 22 in humans) and the *VDJ* locus of the immunoglobulin heavy chain gene (chromosome 14 in humans). According to the current dogma, the insertion of the hypothetical *RAG* transposon into the ancestral vertebrate immune receptor gene happened only once (followed by whole genome duplications) within the timeframe of the V(D)J system appearance. This scenario seems to be unlikely considering that no other traces of *RAG* transposons or their TIRs have been identified elsewhere in higher vertebrate genomes. Furthermore, the presence of *RAG1/2* genes and their possible domestication in invertebrates are considered to have occurred much earlier than their domestication in ancestral jawed vertebrates, for example in purple sea urchin (28).

To address these gaps, we suggest an alternative hypothesis to explain the formation of the V(D)J recombination system in the vertebrate lineage through a more gradual evolutionary process, during which separate *RAG* and *Transib* elements co-evolved to work together. We propose that a prequel to the current RAG recombinase function was its initial domestication for the purpose of host protection. According to the ‘guns for hire’ hypothesis (52) and in similar to domesticated TEs listed above, the RAG1/2 complex, encoded by a domesticated ex-transposon, may have been initially co-opted to protect the host against transposons that could jeopardize the integrity of the genome. As a protein complex with a core that is encoded by a hypothetical immobilized transposon, the ancestral RAG1/2 transposase, with only relatively minor modifications to its amino acid sequence, could have later acquired its current recombinase function.

The current V(D)J recombination process may also be regarded as a failed transposition (**Figure 1A**). A functional and structural analysis of RAG and BbRAGL, revealed a two tier mechanism for domestication and loss of transposition capability in the vertebrate RAG recombinase (63, 64). The first tier is based on a single, highly conserved Arg848 in RAG1. When the RAG1 Arg848 was replaced experimentally with the invertebrate RAG1L equivalent of a Met848, the RAG transposition activity was significantly increased, whereas the reciprocal switch (replacing Met848 in BbRAGL with Arg) had the opposite outcome. The second tier is based on the acidic hinge domain within the first 1–383 aa of vertebrate RAG2. Removing the acidic hinge results in a significant increase in RAG transposition *in vivo*. Together, RAG1 Arg848 and the RAG2 acidic hinge suppress RAG-mediated transposition *in vivo* by more than 1,000 fold (63). We suggest that at least some of these evolutionary changes might have been involved in the initial domestication of the RAG1/2 transposase as the guardian of the genome.

Based on the almost perfect sequence identity between some *Transib* TIRs and the RSSs (**Figure 2B**), we assume that some of the early invading transposons that were recognized by the guardian RAG1/*2* complex were members of the *Transib* family, from which *RAG1* is thought to have originated. As a hypothetical guardian of genome, the early-domesticated RAG1/2 complex may have neutralized the *Transib/Transib-*like transposons by excision and prevention of their reintegration into vertebrate genomes. This notion is consistent with the absence of *Transib* family transposons from all vertebrate genomes that have been analyzed instead of sequenced to the date (8, 9). Following this line of thought, the extant *Ig* family loci with the V(D)J segments are the only places in the genome where the *Transib* transposons survived by purifying selection based on the evolutionary advantage in their specific locations that provided increased immune receptor sequence diversity. How the *Transib* transposons inside *Ig* sequences initially avoided the surveillance of a guardian RAG1/2 is one of the questions that remains open in our hypothesis. It may be that the 12/23 asymmetry of the *Transib* invader has prevented its initial recognition and its full excision from the germline, a malfunction that was later corrected in the contemporary recombination mechanism. The original *Transib* TIR sequences (that became the RSSs) are accordingly the functional descendants of this once neutralized transposon, of which the sequence has been modified to the point of retaining no identifiable similarity to any TE.

Other proteins and enzymes associated with the RAGs during V(D)J recombination may have co-evolved to assist the initial domesticated RAG function as a guardian of the genome. One of these enzymes is TdT, which adds the random N nucleotides to the coding DNA ends after the hairpins are nicked and opened by Artemis/PKc (65). The benefit of this process is the initiation of randomized junctional diversity within the third complementarity-determining region 3 (CDR3) of the Ig and TCR chains that is the key for antigen binding. However, this process is also very wasteful because the junctional diversification process often results in frameshifts that translate into missense or truncated proteins, further supporting the notion that V(D)J recombination was not the original function of the RAG1/2 complex. When considering the RAG complex as a genome guardian, this random sequence diversification process may have been selected for during evolution because it changes the sequence of the original site from which the intervening transposon was excised, providing a protective advantage by preventing its reintegration into the same location in the genome. Supposedly, later in the evolution this junctional diversification process was established to generate immune receptor gene diversity.

The *Ig* gene family structure across different vertebrate species may provide additional clues to the later stages of V(D)J recombination evolution. Although V(D)J recombination is present in all jawed vertebrates, the structure of the *Ig* and *TCR* loci differ significantly among different classes of vertebrates. Two major structures of *Ig*/*TCR* loci are either as a translocon or a cluster. The *Ig* genes in mammals, birds, reptiles, and amphibians bear the more common translocon configuration in which tandem duplications of *V*, *D* (if present) and *J* gene segments are grouped in each locus [reviewed in (15)]. On the contrary, cartilaginous fish such as sharks, skates, and rays have an *Ig* gene cluster configuration in which each cluster is composed of one or two *V* gene segments, one or a few *D* gene segments (if present), a *J* gene segment, and an *Ig* constant region exon. The entire locus is made up of many repeats of such clusters or miniloci (15, 66, 67) where V(D)J recombination occurs within a cluster and not between clusters, resulting in a limited combinatorial diversity (68). Some of the *Ig* clusters in cartilaginous fish contain fully pre-joined (germline-joined) *VJ* or *VDJ* gene segments or partially pre-joined *VD*-*J* combinations indicating the possible activity of RAG machinery in the germline (69, 70). The pre-joined *Ig* genes are expressed during early developmental stages, and thus may provide protection to the progeny of this animal group (71). Few cases of pre-joined *VDJ* segments are present in teleost fish (72) and even in one case of a mammal (73, 74). Therefore, it is plausible that the RAG mechanism may have acted originally in the germline, supporting the suggested hypothetical genome guardian function. Perhaps later in the evolution of the ancestral cartilaginous or of bony fish, this activity became sufficiently advantageous and was shifted to the lymphocyte lineage of the soma (**Figure 3**).

Taken together, we propose that the early evolution of the V(D)J recombination system may have evolved in four gradual steps (**Figure 3**, box). In the first step, an immobilized *RAG1/2* transposon in an ancestral vertebrate was co-opted to guard the genome from other invading TEs. In the second step, a distinct *Transib* transposon with asymmetric TIRs (the Transib seed), was inserted into an antigen receptor gene in an ancestral vertebrate, splitting an exon into *V* and *J* segments. In the third step, the RAG1/2 complex gained its current recombinase function in the germline as has been speculated for some cartilaginous fish. The new feature could have been transferred to the offspring to provide an initial immunological advantage. In the fourth step, the RAG recombination mechanism was selected to function mostly in the somatic lymphocyte cell lineage in higher vertebrates because of the benefits that it imparted to the AIS through *Ig* family sequence diversification.

We suggest that the initial RAG1/2 domestication as a genome guardian might have occurred soon after the origin of the vertebrate lineage. As no substantial evidence exists for our hypothesis, significant experimental work will need to be undertaken to support it. For example, one approach may be to test the possible protective function of the vertebrate RAGs against experimentally induced *Transib* transposon intrusions into the germline genome of model vertebrates such as mouse or zebrafish.

## RAG Domestication in Invertebrates

While the biological functions of the vertebrate RAGs are known, the functions of invertebrate *RAGL* genes are yet unclear. As mentioned above, multiple pairs of *RAG1/2L* and numerous solitary *RAG1L* genes have been identified in invertebrates, including a variety of deuterostomes and even several protostomes (9). The *RAG1/2L* gene pairs in invertebrates are always positioned tail to tail as in vertebrate genomes. They may be incomplete, mutated into pseudogenes, or have the potential to encode intact, full-length proteins (9, 36). The TIRs (RSSs) flanking these gene pairs, when present, have a conserved heptamer CAC sequence, but otherwise show rather weak sequence similarity to the vertebrate RSSs, and none follow the 12/23 spacer asymmetry (**Figure 2C**). Some TIR-bearing *RAG1/2L* gene pairs that have been identified to date are also flanked with TSD sequences adjacent to the TIRs suggesting that they may maintain transposition activity.

Of particular interest are the *SpRAG1L* and *SpRAG2L* gene pair in the purple sea urchin, *S. purpuratus*. The *SpRAG1/2L* genes lack associated TIR sequences but have complete open reading frames, suggesting purifying selection to preserve function. The *SpRAG1/2L* genes produce full-length proteins with all major functional domains similar to the vertebrate RAGs including the RAG1 core with the DDE catalytic center (27, 36), and a complete RAG2 structure including a functional PHD (35). The *SpRAG1/2L* genes are co-expressed during early developmental stages and in immune cells or coelomocytes, and the expressed proteins form a stable complex with each other. Furthermore, SpRAG1L binds to the vertebrate RSSs with 12 bp spacers in the presence of vertebrate high mobility group (HMG) proteins (36). The sea urchin *SpRAG1/2L* genes contain multiple exons, while both vertebrate *RAG* genes consist of a single exon [reviewed in (75)]. The acquisition of introns may enable alternative splicing (76) as was predicted for *SpRAGL* genes (36) that might also point to some kind of functionality. In addition to the *SpRAG1/2L* gene homologs, other purple sea urchin genes, which encode repair enzymes essential in vertebrates for V(D)J recombination, including *Artemis* and *TdT*, are also expressed in adult coelomocytes and some other tissues (77). In other words, sea urchins have all basic components required for a functional vertebrate-like recombination mechanism.

Although the *RAG1/2L* gene pairs among different sea urchin species are located in non-syntenic regions of the genomes (8, 24), they appear to be either silenced or domesticated, and therefore are not likely to transpose. Together, sea urchin RAG1L and RAG2L proteins may form a vertebrate-like RAG1/2 complex that possibly functions as an endonuclease or even as a recombinase of sea urchin immune gene(s), or perhaps has some other unknown function. In the scenario where the sea urchin RAG1/2L serves as a recombinase, given that sea urchin genomes do not have any *V(D)J*-like sequence segments, we hypothesize that the proteins encoded by the *SpRAG1/2L* genes may act on different immunological targets. One of the plausible hypothetical targets may be the *SpTransformer* (*SpTrf*) immune gene family (formerly known as *Sp185/333*) in *S. purpuratus* (78), which appears to be subjected to somatic diversification in individual coelomocytes that includes gene deletions and duplications (79). Interestingly, each of the *SpTrf* genes is surrounded by GA short tandem repeats (80, 81), which may serve as TIR-like signal sequences for RAGL recombination-like activity. The function of the sea urchin RAGL proteins will be noteworthy for future investigations. A crucial step in this direction will be the identification of the genomic targets for RAG1/2L activity in sea urchins.

## Conclusion

In the constant arms race between host and pathogens, an advantageous and permanent invention and refinement of defensive or offensive immune mechanisms is inherently costly. In some cases, it can be a double-edged sword when the immune defense turns to various forms of autoimmunity caused by imperfections and complexities of V(D)J recombination and self/non-self recognition. While the widely accepted transposon/split receptor gene hypothesis may explain many of the molecular adaptations of the RAG complex in its current recombinase function, it has not been clear how the RAGs were selected for this function in the jawed vertebrate lineage. The recruitment of pre-formed alien components of selfish Mobile genetic elements (MGEs) opens the door for understanding the fast and cost-effective incremental improvements in host defense that may have led to the appearance of a new immune diversification mechanism. Accordingly, we postulate that the ancestral vertebrate RAG transposon underwent a domestication process in response to pathogenic TE pressure and became a guardian of the genome against other MGE invasions, which was a prequel to its exaptation for its current recombination function. In contrast to the currently accepted scenario, we suggest that the alien seed that invaded the ancestral *Ig* receptor gene and split it into *V* and *J* segments was not a RAG transposon, but rather a *Transib* transposon with RSS-like asymmetric TIR sequences. This scenario may provide an explanation for the evolutionary forces that gave rise to the V(D)J recombination system.

## Data Availability Statement

Publicly available datasets were analyzed in this study. This data can be found here: http://www.imgt.org.

## Author Contributions

IY, MO, and LCS contributed to the development of the hypothesis. IY, MO, LCS, and JA wrote the article. IY made the figures. All authors contributed to the article and approved the submitted version.

## Conflict of Interest

The authors declare that the research was conducted in the absence of any commercial or financial relationships that could be construed as a potential conflict of interest.

## Publisher’s Note

All claims expressed in this article are solely those of the authors and do not necessarily represent those of their affiliated organizations, or those of the publisher, the editors and the reviewers. Any product that may be evaluated in this article, or claim that may be made by its manufacturer, is not guaranteed or endorsed by the publisher.
